# Exploring all-cause mortality surveillance during the Iberian Peninsula power outage, Spain, 28 April 2025

**DOI:** 10.2807/1560-7917.ES.2025.30.26.2500405

**Published:** 2025-07-03

**Authors:** David García-García, Inmaculada León-Gómez, Lucía Pérez-Marín, Diana Gómez-Barroso

**Affiliations:** 1 National Centre for Epidemiology, Instituto de Salud Carlos III, Madrid, Spain; 2 Epidemiology and Public Health Biomedical Network Research Consortium (CIBERESP), Madrid, Spain; 3 Doctoral Programme in Biomedical Sciences and Public Health, Universidad Nacional de Educación a Distancia (UNED), Madrid, Spain

**Keywords:** Power outage, mortality, surveillance, Iberian Peninsula, Spain

## Abstract

A general power outage affected the Iberian Peninsula on 28 April 2025. We explored all-cause mortality in Spain using the monitoring system MoMo. Starting 28 April, over 3 days, 147 excess deaths (95% CI: − 35 to 330) appeared to occur in the country, corresponding to a 4.2% increase over expected mortality. A larger significant 7.9% increase was estimated in 65–84-year-olds (94 excess deaths; 95% CI: 63 to 125). While MoMo cannot attribute such excesses to specific causes, findings highlight the usefulness of real-time surveillance systems for assessing large-scale emergencies.

An unprecedented general power outage affected the Iberian Peninsula from 12:33 p.m. Central European Time on Monday 28 April 2025 and lasted for ca 10 hours. It mainly affected mainland Spain, mainland Portugal, and Andorra, as well as, to a lesser extent, parts of southern France. More than 50 million people were affected as a result. The outage caused the discontinuation or severe disruption of communications, transport and other essential services. After a gradual recovery process that began in the afternoon, electricity was restored completely in the early hours of 29 April [1,2]. The aim of this study is to examine whether all-cause mortality increased in the days including and following the power outage.

## The mortality monitoring system (MoMo)

The all-cause daily mortality monitoring system (MoMo) [3] was initially implemented in 2004 to monitor excess mortality during the summer after a heatwave had a considerable impact on mortality across Europe in 2003. Over the years, MoMo has evolved to continuously monitor mortality notifications and provide daily national and regional model estimates of expected deaths disaggregated by sex and age group. MoMo has identified the impact (excess mortality, as the difference between modelled estimates and observed mortality) related to heat and cold waves, influenza and COVID-19 [4], as well as other emergencies such as the Madrid train bombings on 11 March 2004 and more recently the 2024 isolated high-altitude depression (or DANA by its Spanish acronym) emergency in Valencia [5].

**Figure 1:**
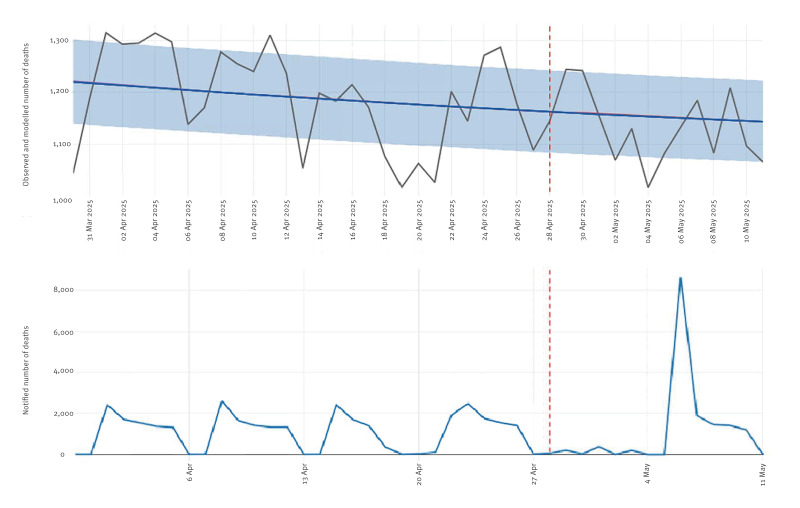
Outputs from the all-cause daily mortality monitoring system, Spain, 30 March–11 May 2025

Using information notified electronically from 4,128 computerised civil registries, certifying deaths across all Spanish provinces and covering 94% of the population, MoMo uses a generalised additive statistical model [6] that handles the inherent delays in notification; currently an average of 4 days occurs between death’s certification and its online submission to MoMo [3]. During the power outage, notifications to MoMo were interrupted with the disconnection lasting for a few days, after which the cumulated delayed notifications were uploaded to the system and regular submission was resumed (Figure 1).

MoMo: mortality monitoring system.

Top: observed mortality (grey line) and model’s estimate for expected mortality (blue line) and corresponding 99% confidence intervals (blue shaded area). Bottom: daily notification count (submission to MoMo may correspond to deaths observed on previous days). Vertical red dashed line marks the day of the power outage (28 April 2025).

## Exploring MoMo all-cause mortality

To identify possible excess in all-cause mortality, we looked at the difference between observed deaths and the model’s estimates for expected deaths on the days following 28 April 2025. To provide context, we compared these differences with a reference historical time period during which mortality could be expected to be similar. This comprised a 4-week period (15 April to 11 May) on the year of the event and 5 additional years, chosen to be 2017, 2018, 2019, 2023 and 2024 in order to exclude atypical fluctuations in mortality due to COVID-19 pandemic [7,8].

**Figure 2:**
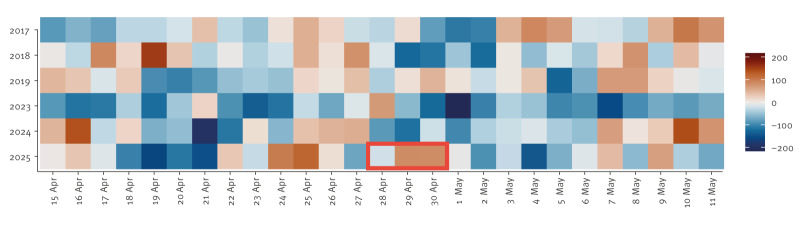
Difference between daily observed and expected deaths in Spain during the reference period: 15 April to 11 May, for years 2017, 2018, 2019, 2023, 2024, 2025

As can be seen in Figure 2, an excess in observed mortality over the expected number of deaths was observed on the 2 days following the power outage (29 April–30 April 2025). More precisely, 84 (95% confidence interval (CI): 23 to 145) and 83 (95% CI: 22 to 144) excess deaths were estimated for each of these 2 days corresponding to a 7.2% (84/1,161 and 83/1,159) increase over expected deaths in both cases. No unusual mortality was observed on the day of the power outage (20 fewer deaths than expected, 1.7% below the estimate). The Table presents more details on the model’s estimates by sex and age group.

Shades of red indicate excess mortality and shades of blue indicate deficit mortality. A red rectangle highlights the day of the power outage and the 2 following days, in which excess mortality was observed.

**Table:** Observed mortality and 3-day cumulative mortality^a^ with for each, the model’s respective estimate, the difference between the observed and the model values, and the relative increase/decrease relative to the model’s estimate, Spain, 28 April 2025

**Population**	**Observed mortality**	**Model’s estimate (95% CI)**	**Excess/deficit mortality (95% CI)**	**Relative increase/decrease (%)**
Total	1,143	1,162 (1,102 to 1,222)	− 20 (− 80 to 40)	− 1.7
Women	588	574 (531 to 617)	14 (− 28 to 56)	2.4
Men	555	588 (545 to 632)	− 34 (− 77 to 10)	− 5.6
0–65-year-olds	164	159 (151 to 167)	6 (− 2 to 14)	3.8
65–84-year-olds	446	453 (443 to 464)	− 7 (− 18 to 3)	− 1.5
≥ 85-year-olds	532	556 (546 to 565)	− 24 (− 33 to 14)	− 4.3
**Population**	**Three-day cumulative observed mortality**	**Model’s estimate (95% CI)**	**Three-day cumulative excess/defect mortality (95% CI)**	**Relative increase/decrease (%)**
Total	3,630	3,483 (3,300 to 3,665)	147 (− 35 to 330)	4.2
Women	1,845	1,720 (1,591 to 1,848)	125 (− 3 to 254)	7.3
Men	1,785	1,763 (1,632 to 1,894)	22 (− 109 to 153)	1.2
0–64-year-olds	481	476 (452 to 500)	5 (− 20 to 29)	1.1
65–84-year-olds	1,453	1,359 (1,328 to 1,390)	94 (63 to 125)	6.9
≥ 85-year-olds	1,696	1,664 (1,637 to 1,691)	32 (5 to 59)	1.9

CI: confidence interval.

^a^ The 3-day cumulative mortality is for 28, 29 and 30 April 2025.

To capture potential lagged effects of the power outage on all-cause mortality, we calculated a 3-day cumulative excess or deficit in mortality by summing the differences between observed and model’s expected deaths on any given day and the 2 subsequent days, for all days included in the reference period. A 3-day cumulative excess of 147 deaths was estimated on 28 April 2025 (4.2% increase over expected deaths, albeit not statistically significant), the twelfth highest of the 162 days included in the reference timespan. As shown in the Table, there appeared to be greater excess deaths for women than men (7.3% increase over expected deaths in women and 1.2% in men). Among all deaths, the excess was significantly greater in individuals aged 65–84 years (6.9% increase over expected deaths in this group) than in any other age groups, including individuals under 65 and over 85 years, for whom less than a 2% increase was found (Table).

**Figure 3:**
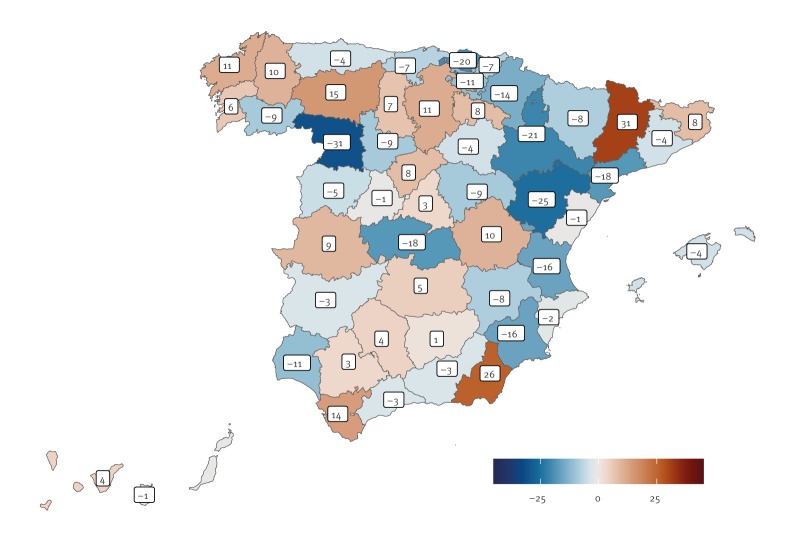
Relative 3-day cumulative excess/deficit mortality (% over/under expected mortality) for each province, Spain, 28 April 2025 (n = 52 provinces)

A heterogeneous spatial distribution was also observed, with some provinces showing higher excesses than others, both in relative terms (Figure 3) and in absolute numbers (see [9] for province-specific estimates).

## Discussion

This report presents a timely assessment of all-cause mortality patterns observed in Spain following the widespread power outage across the Iberian Peninsula on 28 April 2025. The objective was to document anomalies in mortality signals using data from Spain’s MoMo system, as part of its function in real-time epidemiological surveillance.

While a significant mortality excess was observed on 29 and 30 April, it is not clear whether this could be a consequence of the emergency. Ten deaths directly related to the blackout have been reported in the media [10], but it seems hard to determine whether the observed increase in mortality could be due to some extent to indirect consequences of the power outage [11]. Random deviations from the model’s estimates are inherent to the system, and several comparable excesses have been observed in previous years. The observed spatially heterogeneous distribution of excess/deficit mortality, rather than apparent patterns or a uniform common increase, could also be a manifestation of the natural variations in daily mortality.

Lower than expected mortality figures were reported by the European mortality monitoring activity EuroMOMO’s weekly estimate for Spain after the power outage [12]. This deficit is due to the fewer deaths observed in days after 30 April 2025 (Figure 2) and highlights one of the strengths of MoMo: the availability of nearly real-time daily mortality estimates, crucial to detect signals that may be hidden in longer temporal aggregations.

The temporal coincidence of the excess in mortality with the outage could reflect plausible short-term health impacts related to disrupted care, system overload, or reduced resilience in vulnerable populations. However, the main limitation of the MoMo system, and therefore the current study, is that it monitors all-cause mortality and thus cannot establish causality or attribute deaths to specific exposures. A further limitation lies in the fact that the energy supply was restored at different times across various regions of the country — often even within the same municipality — and no official records of these restoration times are available. This also limits the extent of our results, making it difficult to conduct a more detailed analysis that accounts for the impact of the duration of the power outage on mortality at a local level.

Regardless, a notable operational impact of the event was an unprecedented delay in civil registry death notifications to MoMo, briefly interrupting data flow and highlighting system vulnerability during infrastructure shocks. Although this delay did not compromise final counts for the analysed period, it underscores the importance of ensuring resilience in surveillance systems.

## Conclusion

Exceptional events provide opportunities to evaluate nationwide surveillance systems for their aptitude to react to and to detect possible anomalies. We described excess mortality detected through the Spanish MoMo on days after the nationwide power outage in the Iberian Peninsula, as well as the interruption and subsequent resumption of the normal functioning of the system, highlighting the importance of preparedness and resilience in large-scale surveillance systems.

## Data Availability

Data used in the study are publicly available at https://momo.isciii.es/panel_momo/#section-datos.

## References

[1] BOE-A-2025-8486 Orden INT/399/2025, de 28 de abril, por la que se declara la emergencia de interés nacional en el territorio de diversas comunidades autónomas como consecuencia de la interrupción del suministro eléctrico acaecida el 28 de abril de 2025. [BOE-A-2025-8486 Order INT/399/2025, of April 28, declaring the emergency of national interest in the territory of various autonomous communities as a consequence of the interruption of the electricity supply that occurred on April 28, 2025]. Spanish. [Accessed 10 Jun 2025]. Available from: https://www.boe.es/diario_boe/txt.php?id=BOE-A-2025-8486

[2] Interior desactiva la declaración de emergencia de interés nacional en seis comunidades al sobreponerse a las consecuencias del apagón. [The Interior Ministry deactivates the declaration of national interest emergency in six communities as the consequences of the blackout are overcome]. Spanish. [Accessed 10 Jun 2025]. Available from: https://www.lamoncloa.gob.es/serviciosdeprensa/notasprensa/interior/paginas/2025/290425-desactivada-declaracion-emergencia.aspx

[3] MoMo. [Accessed 21 May 2025]. Available from: https://momo.isciii.es/panel_momo/#section-notificaci%C3%B3n

[4] Nørgaard SK Nielsen J Nordholm AC Richter L Chalupka A Sierra NB Excess mortality in Europe coincides with peaks of COVID-19, influenza and respiratory syncytial virus (RSV), November 2023 to February 2024. Euro Surveill. 2024;29(15):2400178. 10.2807/1560-7917.ES.2024.29.15.2400178 38606570 PMC11010589

[5] Leon Gómez IL Pérez-Marín L Gomez-Barroso D. La Importancia del Sistema de Vigilancia de la mortalidad diaria en España (MoMo). Bol Epidemiol Sem (Madr). 2024;32(4):177-8. 10.4321/s2173-92772024000400002

[6] Wood SN. Generalized Additive Models: An Introduction with R, Second Edition. 2nd ed. New York: Chapman and Hall/CRC; 2017. p.496.

[7] León-Gómez I Mazagatos C Delgado-Sanz C Frías L Vega-Piris L Rojas-Benedicto A The Impact of COVID-19 on Mortality in Spain: Monitoring Excess Mortality (MoMo) and the Surveillance of Confirmed COVID-19 Deaths. Viruses. 2021;13(12):2423. 10.3390/v13122423 34960692 PMC8703729

[8] Vestergaard LS Nielsen J Richter L Schmid D Bustos N Braeye T ECDC Public Health Emergency Team for COVID-19. Excess all-cause mortality during the COVID-19 pandemic in Europe - preliminary pooled estimates from the EuroMOMO network, March to April 2020. Euro Surveill. 2020;25(26):2001214. 10.2807/1560-7917.ES.2020.25.26.2001214 32643601 PMC7346364

[9] MoMo. [Accessed 13 Jun 2025]. Available from: https://momo.isciii.es/panel_momo/#section-momo

[10] Asciende a diez el número de muertos causado por el apagón. [The number of deaths caused by the blackout rises to 10]. [Accessed 23 May 2025]. Available from: https://gaceta.es/espana/asciende-a-diez-el-numero-de-muertos-causado-por-el-apagon-masivo-que-afecto-a-toda-espana-20250430-1126/

[11] García Rada A. Spanish hospitals show resilience amid crippling nationwide power outage. BMJ. 2025;389:r867. 10.1136/bmj.r867 40300790

[12] EUROMOMO. [Accessed 24 Jun 2025]. Available from: https://euromomo.eu/

